# Closing the Osteoporosis Care Gap

**DOI:** 10.1007/s11914-020-00644-w

**Published:** 2021-01-09

**Authors:** Kristina E. Åkesson, Fiona E. A. McGuigan

**Affiliations:** 1 Department of Clinical Sciences Malmö, Lund University, Lund, Sweden; 2 Department of Orthopaedics, Skåne University Hospital, 205 02, Malmö, Sweden

**Keywords:** Fracture, Prevention, Fracture liaison service, Frailty, Elderly

## Abstract

**Purpose of Review:**

This review outlines the scope of the problem in osteoporosis care and secondary fracture prevention and describes fracture prevention strategies, with a focus on the frail elderly.

**Recent Findings:**

Despite heightened awareness among patients and clinicians alike and the availability of efficacious anti-osteoporosis medications, osteoporosis is still underdiagnosed and undertreated. However, the introduction of systematic risk assessment and secondary fracture prevention programmes has gained momentum, and evidence of success is accumulating.

**Summary:**

We possess today the knowledge required to close the osteoporosis care gap. The basic components in a secondary prevention model are similar in all health care settings, number one being a dedicated fracture coordinator, with anti-osteoporosis medications and multifaceted falls prevention as cornerstones, particularly in the frailest, both in the near and long-term. Initiation of structured care pathways including the key elements – identification, investigation, intervention and follow-up of adherence – demonstrably reduces re-fracture rates and is cost-effective.

## The Care Gap

As stated in the WHO World Report on Ageing and Health, ‘Today, for the first time in history most people can expect to live into their 60s and beyond’, with the consequence that the old and very old are becoming an increasingly larger proportion of the world population [1].

While many people will live long and healthy lives, for most, the final years are associated with declining health. Fractures are a sign of diminishing musculoskeletal competence, the fracture ultimately resulting in morbidity and mortality. A first fracture is a sentinel event, signalling an increased risk of new fractures, more than doubled by a history of fracture at any site [2, 3] and multiple times in the event of a vertebral fracture [4]. The time frame for new fractures is dependent on fracture type and indirectly on age, since the fracture pattern varies over time. The incidence of the most common first fracture, the distal radius fracture, begins to increase just after menopause in women, followed by vertebral fractures and later, at advanced age, hip fracture.

Regrettably, there are still large gaps in patient care. Despite the awareness of osteoporotic fracture risk and the availability of anti-osteoporosis medications with proven efficacy to reduce fracture rates, osteoporosis is still underdiagnosed and undertreated. This is most obvious in those with the highest risk, those who have already had a fracture. Over the past 10 years, however, the introduction of systematic risk assessment and secondary fracture prevention programmes has gained momentum from the substantial body of evidence demonstrating efficacy.

### Osteoporosis-Related Fractures and Lifetime Risk

**Fig. 1:**
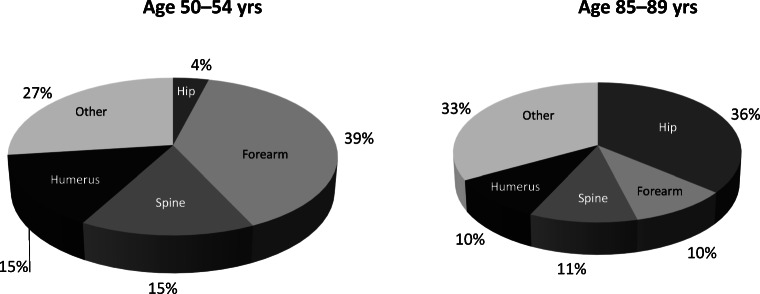
Age- and type-specific fracture pattern

In closing the osteoporosis care gap, a first step is to understand the scope of the problem. Fracture epidemiology is, however, not uniform but related to the geographic and demographic settings. It is also essential to consider other non-modifiable risk factors such as gender, ethnicity and genetics influencing fracture risk in the specific locality. Beyond this, modifiable risk factors are not evenly distributed, with patterns of nutritional intake, smoking and physical activity level being greatly varied not only between countries and continents but also within the local socio-economic environment. Regardless, since age is a determining factor, the distribution between fracture types at various ages has sufficient similarities. Hence, the pattern of osteoporotic fractures is age and site specific: in women between ages 50 and 54 years, fracture of the distal radius is the most common at 39%, with vertebral fractures second at 15% [5]. By ages 85–89 years, hip fractures account for 36%, with distal radius fractures at only 10% (Fig. 1). Acknowledging that prior fracture is one of the strongest risk factors for new fractures, recency, in conjunction with age, exerts enormous influence on time-to-next fracture. For a younger person with a radius fracture, the next fracture may be many years in the future, while for those above 80 years of age, the imminent or short-term risk is high with an up to ten-fold risk [6, 7]. Secondary prevention programmes subsequently have to apply differentiated strategies for relatively younger and older patients to maximize outcome. The majority of osteoporotic fractures are sustained by older persons, and in the following review, the focus will therefore be on the elderly.

## Models of Post-fracture Care

Establishing a secondary prevention programme relies on a number of building blocks that are essentially the same in all settings; however, the implementation and detailed logistics are specific to the local circumstances.

There are two main models of post-fracture care: the *fracture liaison service (FLS)* and *the ortho-geriatric service (OGS).* The fracture liaison service was developed to specifically address the issue of secondary fracture prevention among those having sustained a fracture. The model relies on a fracture coordinator or fracture nurse as the pivotal player. Any fracture related to osteoporosis will be managed within the model, both fractures requiring in-patient care or only out-patient care. FLS programmes commonly include patients above the age of 50 years. The ortho-geriatric service is a branch of geriatrics centred on the older fracture patient, in most cases patients with hip fracture. The approach is more holistic and includes immediate post-fracture management, mobilization, rehabilitation and optimization of health status, in addition to prevention of future fractures in the older persons. Multi-disciplinary teams and co-management are essential requirements within OGS. Since cases are admitted, case finding is given in this model. The challenge is that OGS is not available in most hospitals and hip fracture patients are mainly managed within orthopaedics, where FLS will be the secondary prevention service.

The FLS model is based on four key elements:

- Identification of fracture patients (i.e. case finding)
- Investigation (i.e. risk assessment) with or without bone mineral density measurement
- Intervention with initiation or recommendation of anti-osteoporosis medication, falls prevention and risk factor modification
- Information transfer and follow-up (i.e. adherence to interventions)

Despite seeming simple, it has proven a challenge to initiate post-fracture care programmes that are sustainable. One of the probable difficulties lies in the lack of medical ownership with multiple specialists involved in the fields of orthopaedics, geriatrics and internal medicine including endocrinology or rheumatology, with specialists in this field acting as the osteoporosis specialists, and primary care physicians. Another layer is the crucial multi-disciplinary core of physiotherapists, nurses and pharmacists, while for the oldest, social welfare, home help or institutions are equally important for successful reduction in fracture risk.

The FLS model has been detailed by Ganda et al. using a helpful stratification of efforts [8••]. The type A model is the most comprehensive model and includes identification, risk assessment and treatment initiation. The least intensive model, type D, involves patient education only. Treatment initiation between models differed by 38%, with a comparable increased rate of BMD testing depending on the intensity of the model. Resource allocation is only to some extent related to intensity, while the ability to develop smart logistics adapted to the specific clinical setting is critical, as is a dedicated coordinator. Beyond that, successful services rely on flexible solutions and span primarily within hospital-based models and primary care–based models. The clear implication is that a systematic model, which also initiates treatment, is mandatory if expected treatment targets are to be reached. However, it is also essential to systematically include non-pharmacological interventions and follow-up. The bench-marking programme, the Best Practice Framework, developed by the International Osteoporosis Foundation Capture the Fracture, is one such programme to drive change and improve care by setting standards [9••]. By applying the 13 standards, it is possible to deliver the best possible fracture prevention programme, both for the very elderly with a high imminent risk and for those who are at younger ages where the next fracture may be years ahead. To adapt to the differences in health care systems, other clinical recommendations to facilitate secondary prevention targeting people aged 65 years and older with hip and vertebral fracture are also available [10••]. These ‘hands-on’ guidelines also focus on information transfer to the patient, detailing choice of interventions and how to effectively convey the rationale for interventions to increase compliance.

## Fracture Risk and Recurrence: The Older Patient

All physiological functions decline with age, but arguably, the most dramatic takes place in the musculoskeletal system and is closely linked to frailty – musculoskeletal functioning is a key component for the quantification of frailty; at the same time, frailty is associated with the most common age-related musculoskeletal conditions and fracture. How best to operationalize and measure frailty is debated [11, 12]. Most instruments to measure frailty capture the physical (mobility, strength, physical activity, nutrition and energy), psychological (mood, cognition) and social domains, although the specific assessments within each domain are diverse [12].

The most commonly employed definitions are the frailty phenotype and the frailty index, although as many as 50 exist [13–15]. The frailty phenotype [16] is conceptually simple and operationalized as the presence of 3 out of the following 5 criteria: ‘unintentional weight loss in the preceding 12 months’, ‘self-reported exhaustion’, ‘low grip strength’, ‘slow gait speed’ and ‘low physical activity’. Based on these, individuals can be classified as ‘frail’, ‘pre-frail’, or ‘robust’. The frailty index [14] is based on the accumulation of ‘deficits in health’ and scored from 0.0 to 1.0 based on the number of deficits counted divided by the number included in the index. A higher score indicates higher frailty, and although there is no explicit cut-off defining frailty, an empirical cut-off of ≥ 0.25 has been suggested [17, 18]. Nevertheless, the clinical perception of biological age is equally valuable in a doctor’s office [19].

The elderly with osteoporosis-related fractures should perhaps not be thought of as ‘average elderly’ but rather as frail [20]. In support of this, frailty is higher among those who have suffered a fracture. In a recent study [21], change in frailty status over 12 to 24 months was significantly greater among women sustaining a major osteoporotic fracture. At any age, hip fracture is the most severe, predominantly affecting the already frail. For men, this may be particularly true; typically, although chronologically slightly younger when they attain the fracture, their burden of comorbidities and high early mortality is well-described [22–24] . Post hip fracture, accelerated frailty is a characteristic, and for men and women, mortality continues to be higher than the background population in excess of 10 years post event [25].

The lack of consensus on how to operationalize frailty makes it difficult to reach definitive conclusions on frailty as a predictor of fracture. However, a systematic review and meta-analysis involving 96,564 community-dwelling older men and women (mean age 75–76 years) across 6 studies using the frail and pre-frail classifications has brought some clarity [26]. Frailty was associated with a 70% increased odds risk for a future fracture of any type (pooled OR = 1.70, 95% CI 1.34–2.15, *p* < 0.0001), and even among pre-frail individuals, the odds risk of fracture was increased by 30% (pooled OR = 1.31, 95% CI 1.18–1.46, *p* < 0.0001).

In terms of risk for re-fracture, frailty is largely unexplored as a risk assessment tool, although very likely its assessment would allow personalized interventions for risk reduction and targeted rehabilitation. Post-fracture recovery is dependent on pre-fracture frailty status, and the OGS model should facilitate appropriate care tailored to the needs, resilience and intrinsic capacity of the individuals. Hence, scoring frailty status by whichever instrument, even using the simplest measure, can function as a tool to both assess and follow recovery but also to determine the most appropriate intervention strategy for the individual, definitely in the OGS model, but it would also perfectly complement the FLS.

The ‘vicious cycle’ of functional decline characterizing frailty and fracture and its outcomes is an important consideration in fracture recurrence. Frailty, manifesting in gait problems and weakness in the elderly, contributes to an increased risk of falling, rendering them an important clinical challenge [27]. According to the WHO, 37.3 million falls every year require medical attention of some sort [28], reflecting that falls from a standing height are the leading cause of low-energy fractures in the elderly and the cause of almost all hip fractures [29]. Lost confidence and fear of falling have far-reaching consequences on quality of life [18]. The potential for increased frailty is aptly demonstrated – every third person aged 65 years and older is estimated to suffer at least one fall every year, rising to every second person aged over 80 years.

**Fig. 2:**
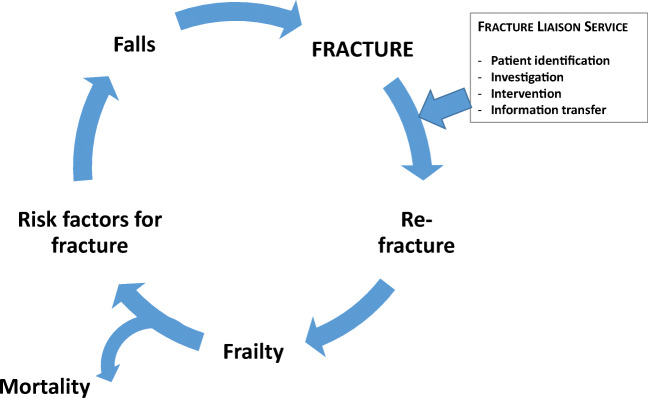
The interaction between risk factors for fracture, falls, frailty, fracture and re-fracture

As a target for secondary fracture prevention, it is a given fact that for the cognitively intact, up to 40% of all falls could be avoided through simple interventions [30]. The majority of longitudinal studies, mostly using the frailty index of deficit accumulation, show the association between increased frailty and increased propensity to fall across short and longer time frames [18, 30, 31]. This highlights the interaction between fracture, clinical risk factors, falls, frailty and recurrence of fracture; each of these requires attention to break the cycle (Fig. 2).

Pharmacological treatment to preserve and increase bone mineral density and reduce fracture risk is the cornerstone of fracture prevention. The association between fracture and bone mineral density is well-described, while cohorts designed to address frailty or other age-related conditions may lack osteoporosis outcomes. While it might not be important for treatment decision in the very elderly fracture patients, frailty might still be an indicator of fracture risk in those who are pre-frail or frail but not yet fractured. In a large cohort of elderly community-dwelling women, indeed, the frailest women did have lower bone density and the highest proportion with osteoporosis. Of note, BMI was greater in those with higher frailty index, presumably due to reduced activity and an overall poorer health [32].

Studies on fracture and frailty in men are more scarce, although a recent large study (*n* = 3231, aged 40–79 years), employing an adapted frailty phenotype and a frailty index, reported a stepwise decrease in calcaneal QUS with increasing frailty [33]. Also, lower BMD was site dependent and influenced by method of frailty assessment. Assessing frailty, particularly in men, may be even more important, since men who attain fracture tend to have underlying comorbidities. Among men as young as 40 years of age with a distal radius fracture, BMD is lower than the population average and osteoporosis three to five times more prevalent [34]. In the future, fracture risk assessment and prevention objectives may be improved by composite definitions of osteoporosis, frailty and sarcopenia adapted to women and men.

### The Economic Burden Associated with Fragility Fractures

Frail individuals require disproportional health care, therefore constituting a larger burden for national health care expenditures across all branches of in- and out-patient health care.

The IOF reports that for 2017, fragility fracture–related costs in the EU were €37.5 billion, surpassing many chronic diseases of old age, while projections indicate an increase of 27% by 2030, with hip fractures accounting for the majority of incurred costs [35]. Globally, the burden is similar. In Asia-Pacific, costs are projected at 25 billion US dollars by 2025 [36] and 95 billion by 2040 in North America [37].

## Post-fracture Programmes: Re-fracture and Survival Outcomes

The ultimate aim of secondary fracture prevention programme is to reduce re-fracture rates. However, additional aims are to improve quality of life by reducing fear of re-fracturing and fear of recurrent falls. Possibly, interventions may also reduce mortality.

A recent systematic literature review (159 publications) and meta-analysis (16 RCTs, 58 observational studies) indicate that re-fracture rates are halved in patients receiving care from an FLS (6.4% versus 13.4%), while risk of re-fracture was lowered by 5% (95% CI – 0.08 to − 0.03) [38].

**Fig. 3:**
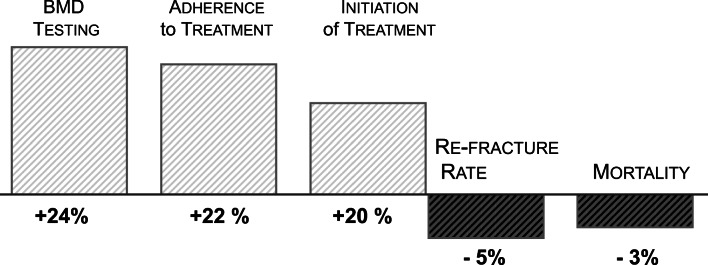
Change in patient outcomes after osteoporosis-related fractures when managed through fracture liaison service (FLS) programmes

In addition to the reductions in fracture associated with FLS, survival is another outcome that is improved. Compared to the average 30-day and 1-year estimates of mortality, Wu et al. also demonstrated that patients participating in an FLS have a significant reduction in mortality (10.4% versus 15.8% in the control arm) in studies with 6–72 months of follow-up (Fig. 3) [38]. Similarly and in a longer time frame, patients followed up to 2 years were also shown to have reduced mortality (35% hazard ratio: 0.65; 95% confidence interval [CI]: 0.53–0.79) [39].

## Evaluating Interventions to Reduce Fracture Recurrence

To evaluate adherence to pharmacological treatment on the individual level has a number of limitations; nonetheless, it is far simpler than obtaining real-world data on non-pharmacological interventions. In a FLS programme, regular follow-up for up to a year is part of the best practice framework [9••]; other possibilities include national databases, registers or claims data. In contrast, evidence for the effectiveness of non-pharmacological interventions relies on clinical trials and subsequent meta-analysis or systematic reviews. Therefore, it is not easy to answer questions on how successful falls education or interventions such as balance and strength training are in FLS or OGS programmes.

Patient adherence to anti-osteoporosis treatment is a challenge, particularly with oral bisphosphonates [40]. Persistence over years is essential to reducing subsequent fractures; hence, discontinuation, which is common, is a substantial problem. Within an FLS, persistence is improved, with as many as 80% still on treatment at 1 year [41, 42]. This contrasts with 40–50% in ‘real-world settings’ [43, 44]. This is also confirmed in the meta-analysis, where adherence to pharmacological treatment was reported to increase by 22 percentage points, and although overall adherence over 3–48 months was lower (57%), this is still superior to the most recent treatment data [38] (Fig. 3). However, even within fracture liaison services, persistence with bisphosphonates after a hip fracture can be as low as 35% among those with poor functional status [45], indicating that additional/personalized strategies are needed for this group. To this extent, the use of annual or bi-annual injectable or infusion dosing, with denosumab or zoledronic acid, respectively, might improve both initiation of therapy and persistence.

For the cognitively intact, up to 40% of all falls could be avoided [30]. A 2019 Cochrane Review indicates that single-intervention exercise programmes can prevent falls in community-dwelling individuals over the age of 60 years. Based on 59 RCTs involving almost 13,000 individuals (predominantly women), exercise reportedly reduced the rate of falls by 23% (rate ratio 0.77, 95% CIs 0.71 to 0.83). To contextualize this, there were 195 (95% CI 144 to 246) fewer falls over 12 months, compared to 850 falls in 1000 controls. Numbers of people falling were 15% lower (risk ratio (RR) 0.85, 95% CI 0.81 to 0.89; 13,518 participants, 63 studies), equating to 72 (95% CI 52 to 91) fewer fallers [46]. Fracture rates related to exercise interventions have also been evaluated in the frail, using Fried’s criteria, with a lower risk for hip fracture, albeit non-significant due to the low number of participants (0.16 (95% 0.01–2.81)) [47]. In community-dwelling women on the other hand, fracture reduction is more substantial (RR 0.39 (95% CI 0.22–0.66)) [48].

More difficult to assess, due to the generally small trial sizes and how the studies were conducted, are single (non-exercise), multifactorial or multi-component interventions. Reflecting this, another Cochrane Review based on 62 RCTs involving almost 20,000 individuals concluded there was *probably* an effect on fall rate, numbers of people falling or risk of falling, but this is very dependent on whether comparison was with an inactive or an exercise-only control [49].

### Intervention Strategies to Reduce or Reverse Frailty

While frailty is dynamic, reversal may be challenging in the frailest. It may be more feasible to preserve the activities of daily living in pre-frail individuals since their response to intervention is better and more intensive interventions are possible. Most interventions focus on nutritional supplementation and resistance training [50–56], with the general agreement that multi-domain interventions are the most effective (such as the OGS), including addressing polypharmacy and social engagement [50, 57]. However, outstanding questions remain, including the duration of intervention.

The economic benefits resulting from intervention strategies to reduce or reverse frailty are few as yet but are likely to be most cost-effective in the frailest [58]. Further studies with larger numbers of individuals and varying frailty statuses across a variety of settings and including fracture outcomes are clearly necessary. Addressing frailty in conjunction with fracture risk will be vital for successful ageing strategies.

## Indicators to Prove Success and Challenges to Implementation

Today, the FLS network is growing, and sustainability is the challenge of the future, requiring the ability to both initiate an FLS programme and to maintain it.

Many FLS programmes have depended on a champion, whose enthusiasm has driven the process, but despite this, assimilation into the regular care pathway within the system has not been possible. Hence, the goal is to reach full integration with the FLS being a standard and routine procedure, independent of a specific supporter. These challenges are played out on the political level, where stakeholders must firstly understand and accept the extent of the problem. The most successful programmes have been able to provide a business case in agreement with the health care provider, highlighting both the human and economic benefits of effective measures to avoid new fractures [59] and, in doing so, have been able to appropriately staff the programme, a major struggle for those starting up.

To provide evidence for continuation, data collection is the most powerful argument, more so if the data are comparable to other sites. Common indicators are warranted and are under development based on the best practice framework and the experience from those more advanced in the implementation of FLS [60–62]. Obvious short-term measures are time from fracture to initiation of therapy, whereas re-fracture rates require longer time periods and are more accessible in countries with developed national registries such as in Scandinavia [63]. Other interventions, such as falls prevention and various training programmes, may have more rapid effects, although it is inherently difficult to capture their efficacy in the everyday clinical setting, since reliable procedure codes, in contrast to diagnostic codes or prescription data, are rarely applied.

To promote a faster rate of progress to integration built on accumulated FLS experience, a mentoring programme, part of the best practice framework, connects experienced sites with those at the early stages of development.

## Future of Secondary Fracture Prevention

It is with some urgency that we must bridge the osteoporosis care gap, particularly in the elderly (whose number is already large) and in anticipation of the, as-yet-unknown, effects on bone health resulting from the more sedentary lifestyles of the younger generations coming through. Estimates from 10 years ago suggest that more than 60% of waking hours are spent in sedentary pursuits [64]. Given the repercussions of not attaining peak bone mass, or maintaining a healthy skeleton in adulthood, the potential for a new and devastating wave of fractures is enormous.

To close the osteoporosis care gap, today, we have the knowledge of what is required. The building blocks are well-described and applicable in all settings. Systematic secondary fracture prevention programmes that are integrated into the care pathway will reach those at highest risk - those who have already had a fracture. These are also the patients who have a very high risk in the short term; hence, timing is imperative. Initiating, perfecting and sustaining a care pathway will improve treatment rates and reduce the recurrence of fracture. The key to success is defining responsibilities and employing a dedicated fracture coordinator for the FLS or dedicated multi-disciplinary team for the ortho-geriatric services. In addition, acceptance and policy change at the highest levels are required from the health care system.

**PRACTICE POINTS**

**Table:** 

• *Systematic and systems integrating secondary fracture prevention programmes such as FLS or ortho-geriatric services improve post-fracture care* • *Dedicated personnel that is specifically assigned is essential to cover the complex interaction between stakeholders from orthopaedics to primary care* • *In the oldest, estimates of frailty may provide important information for osteoporosis, fracture risk assessment and individualized interventions*	

**RESEARCH AGENDA**

**Table:** 

• *Assessment of clinical effectiveness of FLS and OGS in broader settings* • *Use of key performance indicators to compare and improve programmes* • *Analysis of re-fracture rates under FLS and OGS in longer time frames and from national data* • *Identification of the pre-frail at risk of fracture, determining ‘yellow and red flag’ levels for the recurrence of fracture* • *Investigation of the added value of frailty to other risk algorithms such as FRAX or the Garvan Fracture Risk Calculator for the prediction of re-fracture*	

## References

[1] Beard JR, Officer A, de Carvalho IA, Sadana R, Pot AM, Michel JP, Lloyd-Sherlock P, Epping-Jordan JAE, Peeters GMEE(G), Mahanani WR, Thiyagarajan JA, Chatterji S (2016). The world report on ageing and health: a policy framework for healthy ageing. Lancet..

[2] Klotzbuecher CM, Ross PD, Landsman PB, Abbott TA, Berger M (2000). Patients with prior fractures have an increased risk of future fractures: a summary of the literature and statistical synthesis. J Bone Miner Res.

[3] Kanis JA, Johnell O, De Laet C, Johansson H, Oden A, Delmas P (2004). A meta-analysis of previous fracture and subsequent fracture risk. Bone..

[4] Johansson H, Oden A, McCloskey EV, Kanis JA (2014). Mild morphometric vertebral fractures predict vertebral fractures but not non-vertebral fractures. Osteoporos Int.

[5] Johnell O, Kanis JA (2006). An estimate of the worldwide prevalence and disability associated with osteoporotic fractures. Osteoporos Int.

[6] Toth E, Akesson KE, Spångeous A, Ortsater G, Libanati C. Multiple missed opportunities to reduce key fragility fractures: can we afford to continue to ignore the facts. J Bone Miner Res. 2018;32:(Suppl 1). Available at https://www.asbmr.org/meetings/2018-abstracts.

[7] Toth E, Banefelt J, Åkesson K, Spångeus A, Ortsäter G, Libanati C (2020). History of previous fracture and imminent fracture risk in Swedish women aged 55 to 90 years presenting with a fragility fracture. J Bone Miner Res.

[8] Ganda K, Puech M, Chen JS, Speerin R, Bleasel J, Center JR (2013). Models of care for the secondary prevention of osteoporotic fractures: a systematic review and meta-analysis. Osteoporos Int..

[9] Akesson K, Marsh D, Mitchell PJ, McLellan AR, Stenmark J, Pierroz DD (2013). Capture the fracture: a Best Practice Framework and global campaign to break the fragility fracture cycle. Osteoporos Int..

[10] •• Conley RB, Adib G, Adler RA, Akesson KE, Alexander IM, Amenta KC, et al. Secondary fracture prevention: consensus clinical recommendations from a multistakeholder coalition. J Bone Miner Res. 2020;35(1):36–52. **These recommendations highlight the importance of patient communication in order to develop successful implementation of secondary fracture prevention, essential for adherence to treatment.** 10.1002/jbmr.3877 31538675

[11] de Vries NM, Staal JB, van Ravensberg CD, Hobbelen JS, Olde Rikkert MG, Nijhuis-van der Sanden MW (2011). Outcome instruments to measure frailty: a systematic review. Ageing Res Rev.

[12] Theou O, Brothers TD, Mitnitski A, Rockwood K (2013). Operationalization of frailty using eight commonly used scales and comparison of their ability to predict all-cause mortality. J Am Geriatr Soc.

[13] Hyde Z, Flicker L, Almeida OP, Hankey GJ, McCaul KA, Chubb SA (2010). Low free testosterone predicts frailty in older men: the health in men study. J Clin Endocrinol Metab.

[14] Mitnitski AB, Mogilner AJ, Rockwood K (2001). Accumulation of deficits as a proxy measure of aging. Sci World J.

[15] Rockwood K, Song X, MacKnight C, Bergman H, Hogan DB, McDowell I, Mitnitski A (2005). A global clinical measure of fitness and frailty in elderly people. Canadian Med Assoc Journal.

[16] Fried LP, Tangen CM, Walston J, Newman AB, Hirsch C, Gottdiener J, Seeman T, Tracy R, Kop WJ, Burke G, McBurnie MA (2001). Frailty in older adults: evidence for a phenotype. J Gerontol A Biol Sci Med Sci.

[17] Rockwood K, Mitnitski A (2007). Frailty in relation to the accumulation of deficits. J Gerontol A Biol Sci Med Sci.

[18] Kojima G, Kendrick D, Skelton DA, Morris RW, Gawler S, Iliffe S (2015). Frailty predicts short-term incidence of future falls among British community-dwelling older people: a prospective cohort study nested within a randomised controlled trial. BMC Geriatr.

[19] Gerdhem P, Ringsberg K, Akesson K, Obrant KJ (2004). Just one look, and fractures and death can be predicted in elderly ambulatory women. Gerontology..

[20] Roh YH, Noh JH, Gong HS, Baek GH (2017). Effect of low appendicular lean mass, grip strength, and gait speed on the functional outcome after surgery for distal radius fractures. Arch Osteoporos.

[21] Li G, Papaioannou A, Thabane L, Cheng J, Adachi JD (2016). Frailty change and major osteoporotic fracture in the elderly: data from the global longitudinal study of osteoporosis in women 3-year Hamilton cohort. J Bone Miner Res.

[22] Cooper C, Atkinson EJ, Jacobsen SJ, O'Fallon WM, Melton LJ (1993). Population-based study of survival after osteoporotic fractures. Am J Epidemiol.

[23] Kanis JA, Oden A, Johnell O, De Laet C, Jonsson B, Oglesby AK (2003). The components of excess mortality after hip fracture. Bone..

[24] Pande I, Scott DL, O'Neill TW, Pritchard C, Woolf AD, Davis MJ (2006). Quality of life, morbidity, and mortality after low trauma hip fracture in men. Ann Rheum Dis.

[25] von Friesendorff M, McGuigan FE, Wizert A, Rogmark C, Holmberg AH, Woolf AD, et al. Hip fracture, mortality risk, and cause of death over two decades. Osteoporos Int. 2016;27(10):2945–53. 10.1007/s00198-016-3616-5 27172936

[26] Kojima G (2016). Frailty as a predictor of fractures among community-dwelling older people: a systematic review and meta-analysis. Bone..

[27] Clegg A, Young J, Iliffe S, Rikkert MO, Rockwood K (2013). Frailty in elderly people. Lancet..

[28] Forum WW. Innovation for ageing populations – addressing the challenges of frailty and disability 2014. Available from: http://www.who.int/mediacentre/factsheets/fs344/en/. Accessed Dec 2019.

[29] Parkkari J, Kannus P, Palvanen M, Natri A, Vainio J, Aho H, Vuori I, Järvinen M (1999). Majority of hip fractures occur as a result of a fall and impact on the greater trochanter of the femur: a prospective controlled hip fracture study with 206 consecutive patients. Calcif Tissue Int.

[30] Tinetti ME, Speechley M, Ginter SF (1988). Risk factors for falls among elderly persons living in the community. N Engl J Med.

[31] Li G, Ioannidis G, Pickard L, Kennedy C, Papaioannou A, Thabane L, Adachi JD (2014). Frailty index of deficit accumulation and falls: data from the Global Longitudinal Study of Osteoporosis in Women (GLOW) Hamilton cohort. BMC Musculoskelet Disord.

[32] Bartosch P, McGuigan FE, Akesson KE (2018). Progression of frailty and prevalence of osteoporosis in a community cohort of older women-a 10-year longitudinal study. Osteoporos Int.

[33] Cook MJ, Oldroyd A, Pye SR, Ward KA, Gielen E, Ravindrarajah R, Adams JE, Lee DM, Bartfai G, Boonen S, Casanueva F, Forti G, Giwercman A, Han TS, Huhtaniemi IT, Kula K, Lean ME, Pendleton N, Punab M, Vanderschueren D, Wu FC, O'Neill TW, EMAS Study Group (2017). Frailty and bone health in European men. Age Ageing.

[34] Egund L, McGuigan F, Onnby K, Giwercman A, Akesson K (2016). High prevalence of osteoporosis in men with distal radius fracture: a cross-sectional study of 233 men. Calcif Tissue Int.

[35] Broken bones, broken lives: a roadmap to solve the fragility fracture crisis in Europe. Available from: http://share.iofbonehealth.org/EU-6-Material/Reports/IOF%20Report_EU.pdf. Accessed Dec 2019.

[36] Si L, Winzenberg TM, Jiang Q, Chen M, Palmer AJ (2015). Projection of osteoporosis-related fractures and costs in China: 2010-2050. Osteoporos Int.

[37] Lewiecki EM, Ortendahl JD, Vanderpuye-Orgle J, Grauer A, Arellano J, Lemay J, Harmon AL, Broder MS, Singer AJ (2019). Healthcare policy changes in osteoporosis can improve outcomes and reduce costs in the United States. JBMR Plus.

[38] Wu CH, Tu ST, Chang YF, Chan DC, Chien JT, Lin CH, Singh S, Dasari M, Chen JF, Tsai KS (2018). Fracture liaison services improve outcomes of patients with osteoporosis-related fractures: a systematic literature review and meta-analysis. Bone..

[39] Huntjens KM, van Geel TA, van den Bergh JP, van Helden S, Willems P, Winkens B (2014). Fracture liaison service: impact on subsequent nonvertebral fracture incidence and mortality. J Bone Joint Surg Am.

[40] Abrahamsen B (2010). Adverse effects of bisphosphonates. Calcif Tissue Int.

[41] Boudou L, Gerbay B, Chopin F, Ollagnier E, Collet P, Thomas T (2011). Management of osteoporosis in fracture liaison service associated with long-term adherence to treatment. Osteoporos Int.

[42] Eekman DA, van Helden SH, Huisman AM, Verhaar HJ, Bultink IE, Geusens PP (2014). Optimizing fracture prevention: the fracture liaison service, an observational study. Osteoporos Int.

[43] Netelenbos JC, Geusens PP, Ypma G, Buijs SJ (2011). Adherence and profile of non-persistence in patients treated for osteoporosis--a large-scale, long-term retrospective study in the Netherlands. Osteoporos Int.

[44] Kothawala P, Badamgarav E, Ryu S, Miller RM, Halbert RJ (2007). Systematic review and meta-analysis of real-world adherence to drug therapy for osteoporosis. Mayo Clin Proc.

[45] Gamboa A, Duaso E, Marimon P, Sandiumenge M, Escalante E, Lumbreras C (2018). Oral bisphosphonate prescription and non-adherence at 12 months in patients with hip fractures treated in an acute geriatric unit. Osteoporos Int.

[46] Sherrington C, Fairhall NJ, Wallbank GK, Tiedemann A, Michaleff ZA, Howard K, et al. Exercise for preventing falls in older people living in the community. Cochrane Database of Systematic Reviews. 2019;1:Cd012424. 10.1002/14651858.CD012424.pub2 PMC6360922 30703272

[47] Cameron ID, Dyer SM, Panagoda CE, Murray GR, Hill KD, Cumming RG, et al. Interventions for preventing falls in older people in care facilities and hospitals. Cochrane Database of Systematic Reviews. 2018;9:Cd005465. 10.1002/14651858.CD005465.pub4 PMC6148705 30191554

[48] El-Khoury F, Cassou B, Charles MA, Dargent-Molina P (2013). The effect of fall prevention exercise programmes on fall induced injuries in community dwelling older adults: systematic review and meta-analysis of randomised controlled trials. BMJ..

[49] Hopewell S, Adedire O, Copsey BJ, Boniface GJ, Sherrington C, Clemson L (2018). Multifactorial and multiple component interventions for preventing falls in older people living in the community. Cochrane Database of Systematic Reviews..

[50] Morley JE (2013). Frailty, falls, and fractures. J Am Med Dir Assoc.

[51] Daniels R, van Rossum E, de Witte L, Kempen GI, van den Heuvel W (2008). Interventions to prevent disability in frail community-dwelling elderly: a systematic review. BMC Health Serv Res.

[52] Faber MJ, Bosscher RJ, Chin APMJ, van Wieringen PC (2006). Effects of exercise programs on falls and mobility in frail and pre-frail older adults: a multicenter randomized controlled trial. Arch Phys Med Rehabil.

[53] Serra-Prat M, Sist X, Domenich R, Jurado L, Saiz A, Roces A, et al. Effectiveness of an intervention to prevent frailty in pre-frail community-dwelling older people consulting in primary care: a randomised controlled trial. Age Ageing. 2017;1;46(3):401–407. 10.1093/ageing/afw242 28064172

[54] Tikkanen P, Lonnroos E, Sipila S, Nykanen I, Sulkava R, Hartikainen S (2015). Effects of comprehensive geriatric assessment-based individually targeted interventions on mobility of pre-frail and frail community-dwelling older people. Geriatr Gerontol Int.

[55] Milne AC, Potter J, Vivanti A, Avenell A. Protein and energy supplementation in elderly people at risk from malnutrition. Cochrane Database of Systematic Reviews. 2009;15;2009(2):CD003288. 10.1002/14651858.CD003288.pub3 PMC7144819 19370584

[56] Lozano-Montoya I, Correa-Perez A, Abraha I, Soiza RL, Cherubini A, O'Mahony D (2017). Nonpharmacological interventions to treat physical frailty and sarcopenia in older patients: a systematic overview - the SENATOR Project ONTOP Series. Clin Interv Aging.

[57] Fairhall N, Kurrle SE, Sherrington C, Lord SR, Lockwood K, John B, Monaghan N, Howard K, Cameron ID (2015). Effectiveness of a multifactorial intervention on preventing development of frailty in pre-frail older people: study protocol for a randomised controlled trial. BMJ Open.

[58] Fairhall N, Sherrington C, Kurrle SE, Lord SR, Lockwood K, Howard K, Hayes A, Monaghan N, Langron C, Aggar C, Cameron ID (2015). Economic evaluation of a multifactorial, interdisciplinary intervention versus usual care to reduce frailty in frail older people. J Am Med Dir Assoc.

[59] Mitchell PJ, Cooper C, Fujita M, Halbout P, Akesson K, Costa M (2019). Quality improvement initiatives in fragility fracture care and prevention. Current osteoporosis reports.

[60] Judge A, Javaid MK, Leal J, Hawley S, Drew S, Sheard S, et al. Health Services and Delivery Research. Models of care for the delivery of secondary fracture prevention after hip fracture: a health service cost, clinical outcomes and cost-effectiveness study within a region of England. Health Serv Deliv Res. 2016;4(28). 27748091

[61] Javaid MK, Kyer C, Mitchell PJ, Chana J, Moss C, Edwards MH (2015). Effective secondary fracture prevention: implementation of a global benchmarking of clinical quality using the IOF Capture the Fracture(R) Best Practice Framework tool. Osteoporos Int.

[62] Hawley S, Javaid MK, Prieto-Alhambra D, Lippett J, Sheard S, Arden NK, Cooper C, Judge A, REFReSH study group (2016). Clinical effectiveness of orthogeriatric and fracture liaison service models of care for hip fracture patients: population-based longitudinal study. Age Ageing.

[63] Socialstyrelsen SKoL. https://www.socialstyrelsen.se/globalassets/sharepoint-dokument/artikelkatalog/oppna-jamforelser/2018-1-4.pdf 2017. Accessed Dec 2019.

[64] Colley RC, Garriguet D, Janssen I, Craig CL, Clarke J, Tremblay MS (2011). Physical activity of Canadian children and youth: accelerometer results from the 2007 to 2009 Canadian Health Measures Survey. Health Rep.

